# Successful nutritional treatment of superior mesenteric artery syndrome in a non-malnourished patient: a case report

**DOI:** 10.1038/s41430-026-01723-4

**Published:** 2026-03-26

**Authors:** Aurélie Muller, Diane Bechet, Benoit de Courtivron, Aurélie Sabard, Stéphanie Willot, Hubert Lardy, Violette Goetz

**Affiliations:** 1 Paediatrics Departement, CHRU de Tours, Tours, France; 2 Paediatric surgery Departement, CHRU de Tours, Tours, France; 3 Reference center of Tours for inflammatory bowel disease in Children, Tours, France; 4 Reference center of Inborn Errors of Metabolism ToTem, Tours, France

**Keywords:** Nutrition, Paediatrics

## Case Report

Our patient is a 14-year-old boy with severe scoliosis treated by spinal casting, scheduled for spinal arthrodesis. Six months before surgery, he was diagnosed with Crohn’s disease requiring anti-TNF therapy, leading to postponement of the operation. Adalimumab induced deep remission with complete resolution of symptoms and normalization of fecal calprotectin (<30 µg/g) within one year. At age 15, given the sustained remission, anti-TNF therapy was discontinued to perform arthrodesis under optimal safety conditions, as such treatment increases postoperative complications and should be stopped ideally 4 weeks before and after surgery.

**Fig. 1:**
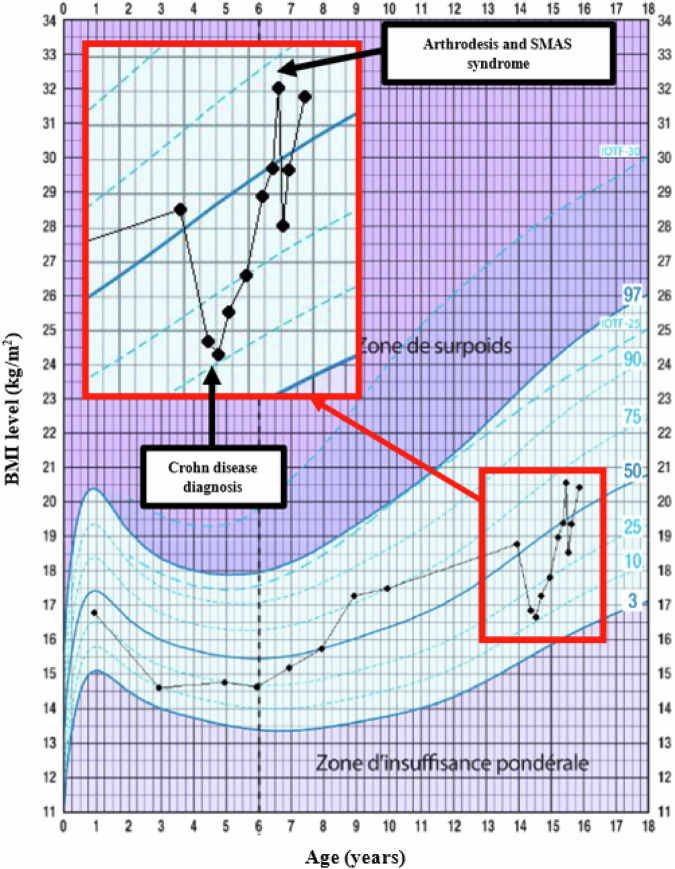
Patient BMI trajectory with focus over the past two years, including the diagnosis of Crohn’s disease and the arthrodesis complicated by SMAS.

During the delay, the spinal curvature slightly progressed, necessitating an extended arthrodesis from T6 to L4. No cranial halo was required. His general condition improved, notably weight gain, and surgery was performed at a normal BMI of 19.2 kg/m² (Fig. 1).

Twenty-four hours postoperatively, the patient developed severe nausea and bilious vomiting. Despite fasting and gradual reintroduction of enteral feeding, each attempt triggered occlusive symptoms (bilious vomiting and abdominal pain). No signs of Crohn’s flare or stenosis were found: no hypoalbuminemia, normal inflammatory markers, rapid postoperative CRP decrease, and normal ferritin (fecal calprotectin could not be measured due to absence of stool). Imaging showed no bowel inflammation. Intermittent macroscopic hematuria was also noted.

**Fig. 2:**
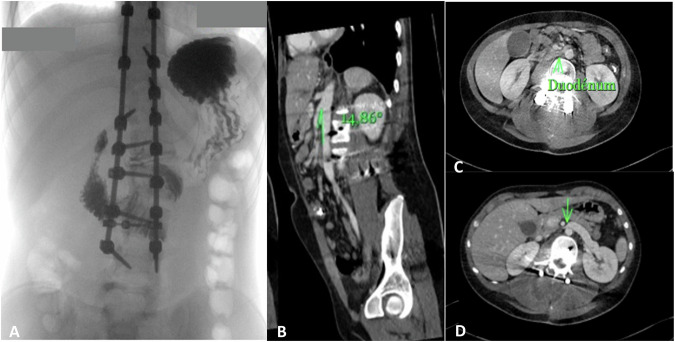
Imaging of the patient confirming superior mesenteric artery syndrome. **A** Upper gastrointestinal contrast showing transient stagnation of contrast in second duodenum. **B** sagittal view of abdominal CT scan revealing the reduction of the arto-mesentérique angle at 14° (normal > 25°). **C** axial view of abdominal CT scan revealing duodenal compression between the mesenteric (in front) and the aortic (behind) artery. **D** Axial view of abdominal CT scan revealing left renal vein compression and upstream expansion.

An abdominal ultrasound on day 5 was normal. On day 11, an upper gastrointestinal contrast study revealed transient contrast stagnation in the second duodenal portion with delayed passage into the third. Contrast-enhanced CT demonstrated compression of the third duodenal segment between the superior mesenteric artery and aorta with a “thread-like” appearance and proximal left renal vein dilatation, consistent with nutcracker syndrome and superior mesenteric artery syndrome (SMAS) (Fig. 2).

A multidisciplinary discussion (pediatric surgeons, orthopedic surgeons, gastroenterologists) favored conservative management, despite the patient’s adequate preoperative nutritional status. Considering the recent complications, resumption of anti-TNF therapy was deferred. Exclusive enteral nutrition (EEN) using **Modulen IBD**^**®,**^ an isocaloric (1 kcal/mL) semi-elemental polymeric formula, commonly used in Crohn’s disease, was initiated. Estimated nutritional requirements were ranged from 2800 and 3100 kcal/day for a 60 kg adolescent with low physical activity, recovering from scoliosis surgery. Enteral feeding was started at 20 mL/h and progressively increased by 10–20 mL/h every 12 h, up to a maximum tolerated rate of 140 mL/h, corresponding to a total daily volume of 2800 mL, with two scheduled 2-h nutrition-free periods. Attempts to increase the infusion rate beyond this threshold resulted in recurrent vomiting.

He was discharged on postoperative day 19 on EEN, having lost 6 kg since admission. Over the following weeks, weight gradually improved. After three weeks, oral liquids were tolerated, followed by mixed and soft foods. Oral caloric intake was assessed weekly by a dietitian, and enteral nutrition was progressively and proportionally reduced to maintain a total caloric intake of 2800v-3100 kcal. Full oral feeding with a low-fiber solid diet, in accordance with Crohn’s disease dietary recommendations, was resumed by six weeks postoperatively, permitting tube removal. Hematuria resolved concurrently. At 8 months follow-up, clinical status remained stable, and weight gain continued with full oral nutrition. The weight monitoring and the dietary regimen adaptation are summarized in Table 1.

**Table 1:** Weight monitoring and evolution of the dietary regimen after the surgery.

	Week 3	Week 4	Week 6	Week 9	Week 20
Weight (kg)	60.2	61.8	63.6	65.5	67.7
BMI (kg/m^2^)	18.7	19.2	19.8	20.2	20.4
Modality of nutrition	EEN		EEN + oral nutrition	Exclusive oral nutrition	
Enteral caloric intake	2800 kcal		Gradual reduction in enteral nutrition proportional to the resumption of oral caloric intake.	/	
Oral food texture	/		Liquid and smooth diet	Low-fiber solid diet	

## Discussion

SMAS also named Wilkie’s syndrome, results from compression of the third duodenal portion between the superior mesenteric artery and aorta, leading to postprandial pain, nausea, vomiting, or complete obstruction. Concomitant compression of the left renal vein may cause microscopic or macroscopic hematuria, known as nutcracker syndrome [1]. Diagnosis relies on imaging, with an aortomesenteric angle <25° and distance <8 mm. CT with sagittal reconstruction is the reference method, while MRA or Doppler ultrasound can also confirm the diagnosis.

SMAS typically develops after rapid weight loss due to loss of mesenteric fat, reducing the aortomesenteric angle. Coexistence with Crohn’s disease is rare and usually secondary to severe disease-related weight loss [2, 3]. Both scoliosis correction surgery and spinal casting can precipitate SMAS by altering intestinal anatomy [4, 5]. The degree of spinal curvature also increases risk [3].

SMAS after scoliosis surgery usually affects malnourished patients, **so** first-line treatment is nutritional -enteral or parenteral- since oral refeeding is often not tolerated. The aim is to restore mesenteric fat and reopen the aortomesenteric angle. Several reports confirm nutritional therapy’s efficacy, though optimal duration remains undefined [6, 7]. When conservative treatment fails, duodenojejunostomy, performed laparoscopically or via laparotomy, is indicated to bypass the obstructed segment [6–8]. Yonggang et al. found that early symptom onset (<48 h post-op) and normal BMI predicts nutritional management failure in SMAS following scoliosis surgery [5].

This case is noteworthy because of the patient’s excellent nutritional status and the very early postoperative onset of symptoms, which made conservative management particularly challenging. However, a surgical approach was highly undesirable as a conservative management strategy of the gastrointestinal tract is generally preferred in pediatric Crohn’s disease. Exclusive enteral nutrition finally allowed symptom resolution and progressive oral refeeding.

At 8 months, the patient remained asymptomatic, with normal diet, weight restoration, and resolution of hematuria. Crohn’s treatment with Adalimumab was safely resumed six weeks after surgery, maintaining deep remission.

## Conclusion

SMAS and nutcracker syndrome are rare but recognized complications following scoliosis surgery. Although they typically occur in malnourished patients with significant weight loss, they may also develop in individuals with a normal BMI, as illustrated in this case. Nevertheless, nutritional management should remain the first-line approach, given its potential for complete recovery and the avoidance of surgical intervention, particularly in patients with comorbidities such as Crohn’s disease.
